# Relapsing Remitting Multiple Sclerosis in X-Linked Charcot-Marie-Tooth Disease with Central Nervous System Involvement

**DOI:** 10.1155/2015/841897

**Published:** 2015-03-25

**Authors:** Georgios Koutsis, Georgia Karadima, Paraskewi Floroskoufi, Maria Raftopoulou, Marios Panas

**Affiliations:** Neurogenetics Unit, 1st Department of Neurology, University of Athens Medical School, Eginition Hospital, 74 Vasilissis Sophias Avenue, 11528 Athens, Greece

## Abstract

We report a patient with relapsing remitting multiple sclerosis (MS) and X-linked Charcot-Marie-Tooth disease (CMTX), carrying a GJB1 mutation affecting connexin-32 (c.191G>A, p. Cys64Tyr) which was recently reported by our group. This is the third case report of a patient with CMTX developing MS, but it is unique in the fact that other family members carrying the same mutation were found to have asymptomatic central nervous system (CNS) involvement (diffuse white matter hyperintensity on brain MRI and extensor plantars). Although this may be a chance association, the increasing number of cases with CMTX and MS, especially with mutations involving the CNS, may imply some causative effect and provide insights into MS pathogenesis.

## 1. Introduction

X-linked Charcot-Marie-Tooth disease (CMTX) is a hereditary neuropathy caused by mutations in* GJB1 *coding for connexin-32 (Cx32), a gap-junction protein expressed in peripheral Schwann cells and oligodendrocytes within the central nervous system (CNS) [1]. Subclinical CNS involvement documented on brain MRI is not uncommon in patients with CMTX. Less commonly, clinical involvement ranging from extensor plantars to acute transient encephalopathy can be observed [1–4]. To date, there have been two reports of patients with CMTX that developed CNS demyelinating disease compatible with the diagnosis of multiple sclerosis (MS) [5, 6].

## 2. Case Report

**Figure 1:**
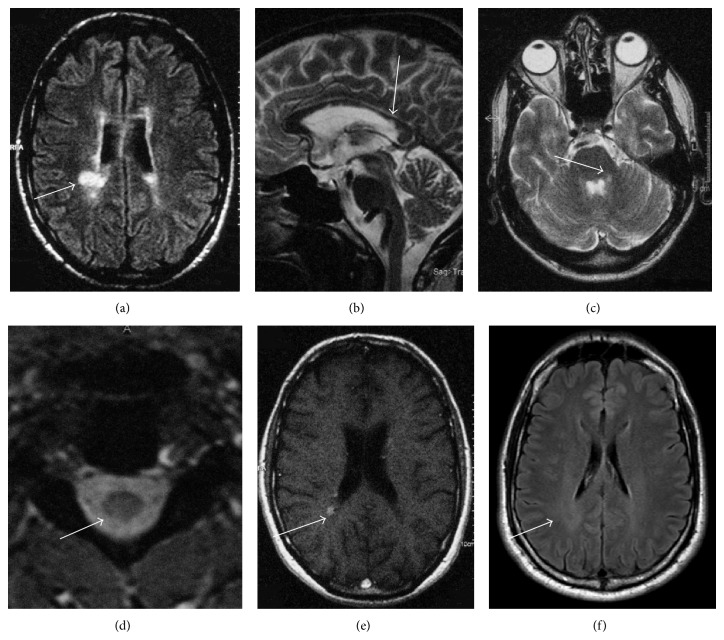
Brain and spinal cord MRI of the patient with CMTX and MS and brain MRI of his first cousin with CMTX and asymptomatic diffuse CNS involvement. (a) Axial FLAIR brain MRI of the patient demonstrating periventricular lesions characteristic of MS. (b) Sagittal T2 brain MRI of the patient revealing lesions in the corpus callosum characteristic of MS. (c) Axial T2 brain MRI of the patient demonstrating a lesion in the left middle cerebellar peduncle. (d) Axial T2 cervical cord MRI of the patient demonstrating a spinal cord lesion. (e) Axial T1 with IV contrast brain MRI of the patient showing periventricular gadolinium-enhancing lesions suggesting inflammation. (f) Axial FLAIR brain MRI of the patient's first cousin revealing diffuse white matter hyperintensity more prominent posteriorly.

We present a case of a 52-year-old man diagnosed with MS five years previously, who had a family history of peripheral neuropathy with no male-to-male transmission. He reported pes cavus since childhood, but no other significant early symptoms. Nerve conduction studies confirmed the presence of a hereditary neuropathy with intermediate velocities. At age 46, he developed left-sided optic neuritis (ON). Brain MRI revealed multiple focal periventricular, callosal, and brainstem lesions, some gadolinium-enhancing, strongly suggestive of MS (Figures 1(a), 1(b), 1(c), and 1(e)). Visual evoked potentials were prolonged ipsilaterally. CSF analysis revealed no oligoclonal bands (OCB) and normal IgG index. The ON remitted following corticosteroid treatment but relapsed a few months later and was further treated successfully with steroids. Later that year, the patient developed gait unsteadiness, with further MRI evidence of disease activity. This remitted with corticosteroid treatment. He was then started on prophylactic treatment with beta-interferon. Two years later, he had an episode of numbness affecting the extremities. Spinal cord MRI revealed a cervical T2 lesion compatible with demyelination (Figure 1(d)). The episode was treated successfully with corticosteroids. Following a further relapse with vertigo and gait unsteadiness, combined with MRI evidence of disease activity, he was switched to natalizumab and has remained relapse-free since. On examination, he had difficulty with tandem gait, saccadic pursuit, extensor plantars, brisk upper limb tendon reflexes, reduced knee jerks, absent Achilles reflexes, mild peripheral atrophy in upper and lower limbs, reduced vibration sense peripherally in the lower limbs, pes cavus, and hammer toes. The patient came to our attention after examining other members of his family who had CMTX and evidence of asymptomatic CNS involvement on brain MRI (diffuse white matter hyperintensity, Figure 1(f); patient's first cousin) or clinically (extensor plantars; patient's aunt). More specifically, brain MRI of the patient's cousin revealed nonenhancing, symmetrical, diffuse white matter abnormalities, which were clearly detectable at the level of the centrum semiovale and periventricularly, were more prominent posteriorly, and involved the splenium of the corpus callosum, as previously described [7]. There were no focal lesions suggestive of inflammatory demyelination, in contrast to the patient. Both relatives were found to carry a novel c.191G>A point mutation in* GJB1* (p. Cys64Tyr) [7]. This mutation was subsequently also identified in our patient with MS.

## 3. Discussion

This is the third patient with CMTX that developed a demyelinating illness fulfilling the diagnostic criteria for MS [8]. Another patient has been described, who had brain MRI evidence suggestive of MS and positive intrathecal OCB and IgG index, but no symptoms or signs suggestive of CNS demyelination [2]. Previously reported symptomatic patients had relapsing forms of MS with MRI evidence of inflammatory demyelination and intrathecal OCB. No evidence of asymptomatic CNS involvement in other family members with CMTX was presented in these publications [5, 6]. This is therefore a unique and interesting feature of the present report. Brain MRI of the patient's cousin showed symmetrical diffuse white matter abnormalities, which have been previously described in CMTX, but no evidence of focal, inflammatory demyelination [4, 7]. It is noteworthy that the distribution of focal inflammatory lesions observed in the patient shows some anatomical correspondence to the diffuse changes seen in his cousin.

A superimposed inflammatory demyelinating neuropathy has been described in patients with CMT1A and CMTX and is probably more frequent than expected by chance [9]. This observation suggests that inherited peripheral demyelination may trigger an autoimmune reaction against peripheral myelin. It is intriguing to hypothesize an equivalent phenomenon directed against CNS myelin in families carrying Cx32 mutations that appear to involve the CNS white matter. The mechanism may include the exposure of myelin antigens to which central tolerance has not been induced, which can then be presented to autoreactive T-cells and trigger an autoimmune reaction against CNS myelin [10].

## 4. Conclusion

We report a patient with CMTX who developed typical relapsing remitting MS. This case is unique in the fact that other family members carrying the same mutation were found to have CNS involvement, with diffuse white matter hyperintensity on brain MRI. The increasing number of cases with CMTX and MS, especially with mutations involving the CNS, may imply some association and provide insights into MS pathogenesis.

## References

[1] Kleopa K. A., Scherer S. S. (2006). Molecular genetics of X-linked charcot-marie-tooth disease. *NeuroMolecular Medicine*.

[2] Panas M., Karadimas C., Avramopoulos D., Vassilopoulos D. (1998). Central nervous system involvement in four patients with Charcot-Marie-Tooth disease with connexin 32 extracellular mutations. *Journal of Neurology, Neurosurgery, and Psychiatry*.

[3] Panas M., Kalfakis N., Karadimas C., Vassilopoulos D. (2001). Episodes of generalized weakness in two sibs with the C164T mutation of the connexin 32 gene. *Neurology*.

[4] Paulson H. L., Garbern J. Y., Hoban T. F. (2002). Transient central nervous system white matter abnormality in X-linked Charcot-Marie-Tooth disease. *Annals of Neurology*.

[5] Isoardo G., Di Vito N., Nobile M., Benetton G., Fassio F. (2005). X-linked Charcot-Marie-Tooth disease and progressive-relapsing central demyelinating disease. *Neurology*.

[6] Parman Y., Ciftci F., Poyraz M. (2007). X-linked Charcot-Marie-Tooth disease and multiple sclerosis. *Journal of Neurology*.

[7] Karadima G., Koutsis G., Raftopoulou M., Floroskufi P., Karletidi K.-M., Panas M. (2014). Four novel connexin 32 mutations in X-linked Charcot-Marie-Tooth disease. Phenotypic variability and central nervous system involvement. *Journal of the Neurological Sciences*.

[8] Polman C. H., Reingold S. C., Banwell B. (2011). Diagnostic criteria for multiple sclerosis: 2010 Revisions to the McDonald criteria. *Annals of Neurology*.

[9] Ginsberg L., Malik O., Kenton A. R. (2004). Coexistent hereditary and inflammatory neuropathy. *Brain*.

[10] Sallusto F., Impellizzieri D., Basso C. (2012). T-cell trafficking in the central nervous system. *Immunological Reviews*.

